# Machine learning for endoscopic third ventriculostomy success prediction—a systematic review and meta-analysis

**DOI:** 10.1007/s00381-025-06962-7

**Published:** 2025-09-29

**Authors:** Anna Łajczak, Yasmin Picanço Silva, Paweł Łajczak

**Affiliations:** 1 Department of Biophysics, Faculty of Medical Sciences in Zabrze, Medical University of Silesia in Katowice, Katowice, Poland; 2 Healthcare Institution of South Iceland, Selfoss, Iceland

**Keywords:** Machine learning, Endoscopic third ventriculostomy, ETVSS, Artificial intelligence

## Abstract

**Background:**

Endoscopic third ventriculostomy (ETV) is a common treatment for pediatric obstructive hydrocephalus, but predicting its success remains challenging. Traditional predictive tools, such as the Endoscopic Third Ventriculostomy Success Score (ETVSS) and logistic regression (LR) models, are widely used; however, recent advancements in machine learning (ML) have shown promise in improving prediction accuracy. This systematic review and meta-analysis aim to evaluate the effectiveness of ML models in predicting ETV success and compare them to traditional models.

**Methods:**

A systematic search across five databases was performed. Authors searched for studies, which used ML algorithms to predict ETV success. This review included studies included studies that reported the area under the receiver operating characteristic curve (AUC) for model performance. ETV success was considered the absence of ETV failure criteria in 6 months after procedure: either recurrence of hydrocephalus symptoms, repeated surgery, or mortality.

**Results:**

A total of four studies involving 3087 pediatric patients were included. The overall pooled AUC for ML models was 0.63 (95% CI 0.56–0.70), with significant heterogeneity (I^2^ = 96%). Subgroup analysis revealed that models including imaging data had a slightly higher AUC (0.74, 95% CI 0.61–0.88). No significant differences were found between ML models and traditional tools like ETVSS or LR models.

**Conclusions:**

ML models show moderate potential for predicting ETV success but do not outperform traditional tools like ETVSS and LR models in clinical application. High heterogeneity and methodological limitations suggest that further research is needed to optimize and validate ML algorithms.

**Supplementary information:**

The online version contains supplementary material available at 10.1007/s00381-025-06962-7.

## Introduction

 Endoscopic third ventriculostomy (ETV) is a widely used surgical procedure for treating obstructive hydrocephalus, a condition affecting around 1 in 500 children [1]. In this procedure, a small hole is created in the floor of the third ventricle to allow cerebrospinal fluid to bypass the obstruction. Minta and colleagues, in their meta-analysis, pooled a mean 81.8% success rate for pediatric hydrocephalus ETV treatment [2]. The success of this procedure is crucial, as it can significantly improve patient outcomes and reduce the need for shunt placement.

However, predicting the procedure’s success has been challenging, depending on various patient-specific factors, surgical techniques, and postoperative management [3–5]. Researchers have developed the Endoscopic Third Ventriculostomy Success Score (ETVSS), a predictive tool that can estimate the likelihood of ETV success based on patient characteristics. This tool has been extensively studied and validated [6]. While promising, various studies observed that the ETVSS tool tends to underestimate the actual success rate prediction [7, 8]. For example, Foley and colleagues evaluated the effectiveness of ETVSS on the Irish population and achieved an area under the ROC curve (AUC) of 0.61 (95% CI 0.49–0.71) [8]. Furtado et al. performed external validation of the ETVSS among Brazilian patients, achieving an AUC of 0.660 at 6 months and 0.668 at 1 year. Labidi et al. evaluated ETVSS in the Canadian population, including adults and pediatric patients, achieving an AUC of 0.61 [9].

The aforementioned observational studies showed, at most, a moderate effectiveness of ETVSS. The need for better predictor models led to the development of logistic regression (LR) models in the literature [10]. Furthermore, recent advancements in machine learning (ML) have demonstrated promise in creating predictive models for various medical applications, such as postoperative complications and risk stratification [11–13]. These advanced methods can analyze extensive patient data, encompassing demographic, clinical, and intraoperative factors, to identify patterns and develop predictive models [12]. However, while ML incorporates advanced computer technologies and presents effectiveness superior to traditional prognostic models in various areas of medicine, the systematic synthesis of current literature is notably absent [14–16].

In this systematic review and meta-analysis, authors aim to evaluate the current state of ML in predicting the success of ETV. This meta-analysis aims to describe the effectiveness, advantages, and limitations of current models. Additionally, authors plan to compare ML models to traditional ETVSS and LR models.

## Methods

This systematic review followed the Preferred Reporting Items for Systematic Reviews and Meta-Analyses (PRISMA) statement and the Cochrane Handbook for Systematic Reviews of Interventions [17, 18].

### Eligibility criteria

This review included studies if they followed inclusion criteria:

1. Included patients undergoing ETV (both rigid and flexible endoscopes considered); and
2. Used advanced ML models to predict ETV success—these algorithms include, for example, naïve Bayes (NB), random forest (RF), eXtreme Gradient Boosting (XGBoost), artificial neural network (ANN), support vector machine (SVM), convolutional neural networks (CNN), deep learning algorithms (DL), and reinforcement learning (RL); and
3. Reported effectiveness of the prediction in the area under the ROC curve (AUC) value. Authors excluded abstract-only works, editorials, review papers, and other non-primary studies.

Furthermore, non-English studies were translated where necessary.

Logistic regression (LR) and ETVSS algorithms were used as a control and compared in a secondary analysis.

### Search strategy and screening

Five databases—PubMed, Embase, Cochrane Library, Scopus, and Web of Science—were searched for eligible articles from inception to December 2024. Keywords in the search included: “Endoscopic Third Ventriculostomy Success Score,” “ETV,” “ETVSS,” “Endoscopic Third Ventriculostomy,” “artificial intelligence,” “convolutional neural network,” “CNN,” “deep learning,” “machine learning,” “decision tree,” “neural network,” “support vector machine,” “k-means,” “artificial neural,” “reinforcement learning.” A detailed search string with Boolean operators is available in the Supplementary Material. Authors imported database files into Covidence, and duplicate entries were removed with software assistance. Both title/abstract and full-text screening were performed by two authors independently, with conflicts resolved by mutual agreement with all reviewers.

### Data extraction

Data extraction was performed independently by two authors. Extracted data included ML models used, comparators (LR, ETVSS, and/or other), validation method of the algorithm (split, cross-validation), dataset splitting (proportion of data for training/validation/testing), number of (female) patients, age, BMI, hydrocephalus etiology (aqueductal stenosis, midbrain tumor/lesion, post-infectious, post-prematurity-related intraventricular hemorrhage, myelomeningocele, non-tectal brain tumor, or other), hydrocephalus type (communicating, noncommunicating), previous shunt, number of patients in training/validation/testing sets, ETVSS, choroid plexus cauterization (none, partial unilateral, complete bilateral), and mortality.

### Outcomes

ETV success was considered the absence of ETV failure criteria within 6 months after procedure: either recurrence of hydrocephalus symptoms, repeated surgery, or mortality.

The effectiveness of the models was assessed with the area under the ROC curve (AUC) [19]. AUC ranges from 0 to 1—a value closer to 1 indicates better prediction performance, while a value closer to 0 is worse. An AUC of 0.5 indicates random guessing. The thresholds for AUC interpretation vary in the literature, but generally, Ań AUC below 0.6 is considered very poor, between 0.6 and 0.7 weak, 0.7 to 0.8 moderate, 0.8 to 0.9 high, and between 0.9 and 1 excellent [20]. Authors performed a single-arm analysis to assess the effectiveness of ML models and a separate analysis to analyze only the most effective ML model from each study. Each model was treated as a separate, independent study.

Secondly, authors evaluated the effectiveness of ML models to ETVSS and LR models in a pairwise meta-analysis.

### Quality assessment

Authors used the Risk Of Bias In Non-randomized Studies-of Interventions (ROBINS-I) tool to assess the risk of bias across seven domains [21]. These domains included confounding, participant selection, classification, deviations from interventions, missing data, selection of reported results, and their measurement. Authors performed a quality assessment process by two authors independently. Results of the quality assessment were visualized with a traffic light plot in robvis [22].

### Statistical approach

Authors applied R software with the meta and dmetar packages [23, 24]. A random-effects inverse variance (IV) model was used for analyses. A single-arm meta-analysis was reported with AUC and ninety-five percent confidence intervals (95% CI). Pairwise meta-analysis was reported as mean difference (MD) with 95% CI; a *p*-value < 0.05 was considered significant. The mean difference of the AUC values was estimated using the methods by Hanley et al. for independent ROC curves [25, 26]. The results of the analyses were visualized with forest plots.

The heterogeneity of the results was assessed with I^2^, and I^2^ > 50% was considered high heterogeneity. Due to a limited number of studies, heterogeneity exploration analyses were pooled only for the overall analysis. Authors performed subgroup analyses (inclusion of imaging variables, ML method), leave-one-out analysis, Baujat plots, influence diagnostics analysis, and funnel plots with Egger’s test (*p* < 0.05 was considered significant evidence for publication bias).

## Results

**Fig. 1:**
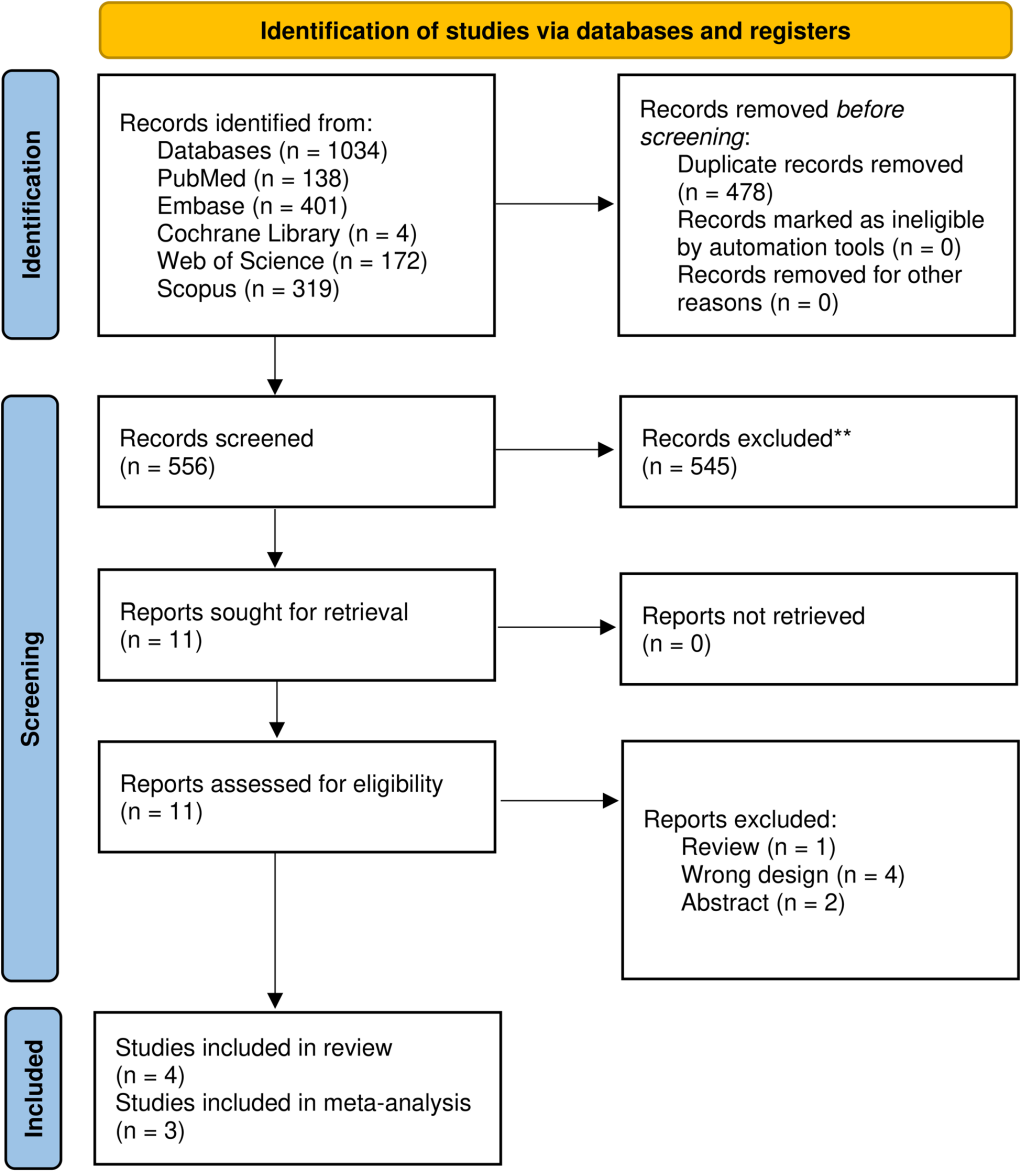
PRISMA flow diagram

Authors searched five databases and found 1034 records: 138 from PubMed, 401 from Embase, 4 from Cochrane Library, 172 from Web of Science, and 319 from Scopus. With the help of software, authors removed 478 duplicate entries before the title/abstract screening. Eleven articles underwent full-text screening; two abstract papers, one review, and four studies with the wrong design and aim were removed. A total of four articles were finally included [27–30]. The detailed search process is visualized in Fig. 1.

### Characteristics of included studies

Four studies were included—two from Iran, one from a US-Canadian collaboration, and one solely from the USA. A total of 3087 patients were analyzed—1995 patients had successful ETV (64.6%), and 1029 had ETV failure (35.4%). Hydrocephalus etiologies included other (37.7%), aqueductal stenosis (19.8%), midbrain tumor/lesion (21.5%), post-infectious (2.3%), post-prematurity-related intraventricular hemorrhage (6.0%), myelomeningocele (5.4%), and non-tectal brain tumor (7.3%). ML models included ANN (3), XGBoost (2), SVM (2), NB (2), RF (1), and k-NN (1). Fivefold cross-validation was used in three studies, while split once. Tables 1 and 2 describe the full study characteristics.

**Table 1:** Baseline details. S - Success; F - Failure

Study	ML model (s)	Comparator	Validation	Dataset splitting	Number of patients		Female		Age		BMI	
					S	F	S	F	S	F	S	F
Malhotra et al. [27]	XGBoost, SVM, ANN, NB	ETVSS	Fivefold cross-validation	70% training/30% testing	567	185	245	92	> 10 years: 240; 1–10 years 252; 6–12 months 30; 1–6 months 27; < 1 month 18	> 10 years: 49; 1–10 years 73; 6–12 months 22; 1–6 months 23; < 1 month 18	N/A	
Azimi et al. [28]	ANN	LR, ETVSS, CURE Children’s Hospital of Uganda (CCHU) ETV Success Score	Split	50% training/25% validation/25% testing	79	89	41	47	2.2 ± 1.8	0.7 ± 1.1	14.5 ± 13.1	11.2 ± 9.9
Adil et al. [29]	SVM, RF, NB, XGBoost, k-NN	LR, ETVSS	Nested fivefold cross-validation	85% training/15% testing	1261	786	542	337	> 10 years: 448; 1–10 years 539; 6–12 months 90; 1–6 months 121; < 1 month 63	> 10 years: 166; 1–10 years 276; 6–12 months 142; 1–6 months 148; < 1 month 54	N/A	
Masoudi et al. [30]	ANN	ETVSS	Fivefold cross-validation	70% training/30% testing	88	32	N/A		N/A		N/A	
Study	Hydrocephalus Etiology											
	Aqueductal stenosis		Midbrain tumor/lesion		Post-infectious		Post prematurity-related intraventricular hemorrhage		Myelomeningocele		Non-tectal brain tumor	
	S	F	S	F	S	F	S	F	S	F	S	F
Malhotra et al. [27]	115	33	189	42	12	6	9	12	13	8	94	31
Azimi et al. [28]	27	10	6	4	4	8	13	10	11	10	7	6
Adil et al. [29]	252	150	294	104	18	20	56	77	72	47	56	22
Masoudi et al. [30]	N/A											
Study	Hydrocephalus Etiology						Hydrocephalus type					
	Other						Communicating			Non-communicating		
	S			F			S		F	S	F	
Malhotra et al. [27]	135			53			N/A			N/A		
Azimi et al. [28]	11			41			19		34	60	55	
Adil et al. [29]	513			366			N/A			N/A		
Masoudi et al. [30]	N/A						N/A			N/A

**Table 2:** Supplementary information from the studies. S - Success; F - Failure

Study	Previous shunt		Training size		Validation size		Testing size		ETVSS																		Choroid plexus cauterization						Mortality	
									10		20		30		40		50		60		70		80		90		None		Partial unilateral		Complete bilateral			
	S	F	S	F	S	F	S	F	S	F	S	F	S	F	S	F	S	F	S	F	S	F	S	F	S	F	S	F	S	F	S	F	S	F
Malhotra et al. [27]	0	0	397	127	Not performed	Not performed	170	58	0	3	1	0	1	5	21	16	28	20	11	3	82	45	235	55	188	38	0	0	0	0	0	0	N/A	N/A
Azimi et al. [28]	10	26	84		42		42		N/A						≤ 40: 12	≤ 40: 57	50–70: 48	50–70: 29	N/A				≥ 80: 19/3	≥ 80: 19/3	N/A		34	65	5	4	40	20	N/A	N/A
Adil et al. [29]	297	287	N/A		N/A		N/A		N/A																		N/A						11	6
Masoudi et al. [30]	N/A		N/A		N/A		N/A		N/A																		N/A						N/A	

### Risk of bias

**Fig. 2:**
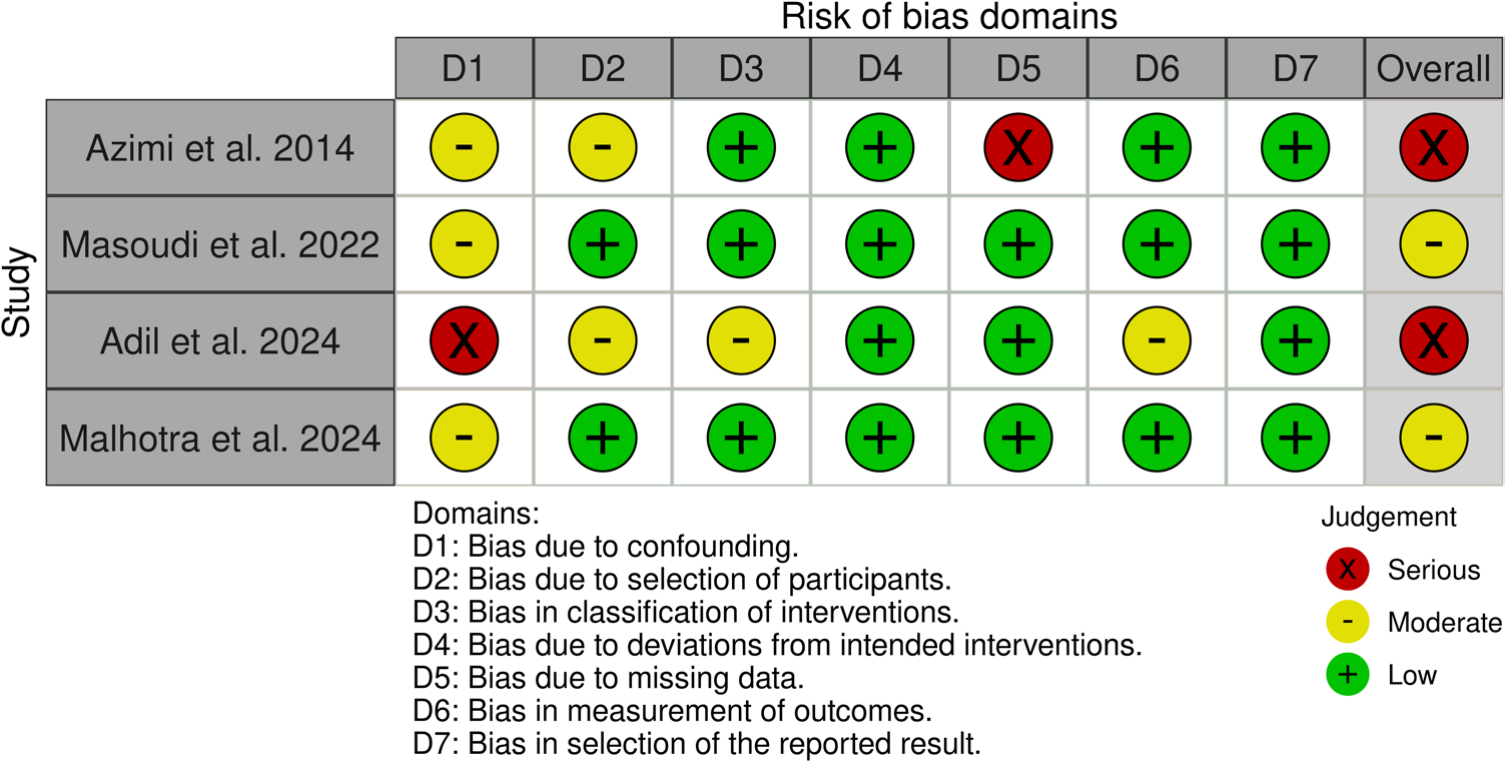
Risk of bias

None of the studies was assessed with a low risk of bias. Most of the bias was found in the confounding domain; however, concerns in participant selection, intervention classification, missing data, and outcome reporting were found. Azimi et al. and Adil et al. studies were assessed with a high risk of bias, while Masoundi et al. and Malhotra et al. were assessed with a moderate risk of bias. Figure 2 is a traffic plot with risk of bias (ROBINS-I) results.

### Meta-analysis

**Fig. 3:**
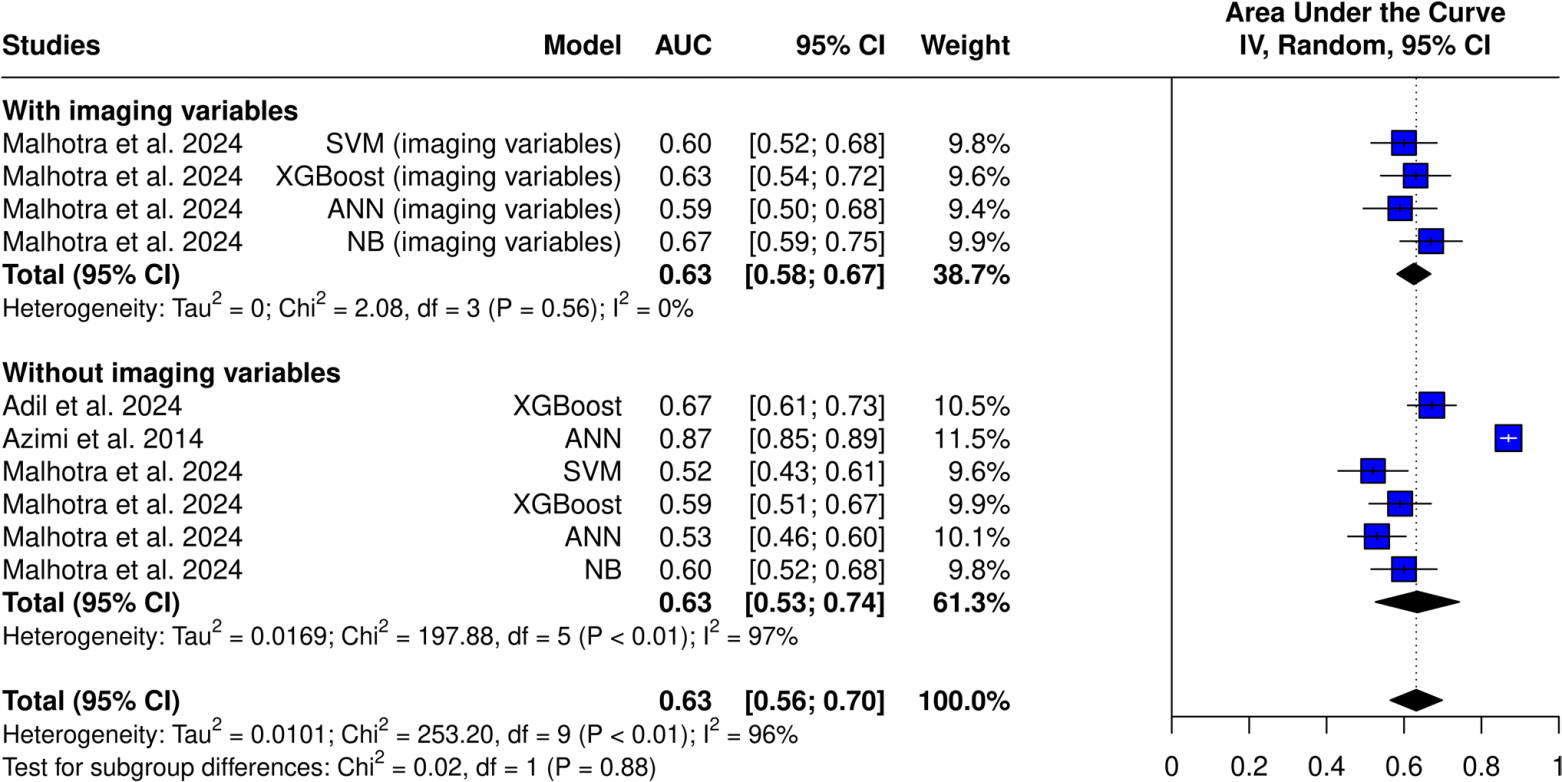
Overall pooled AUC from all ML models (imaging variable subgroups)

**Fig. 4:**
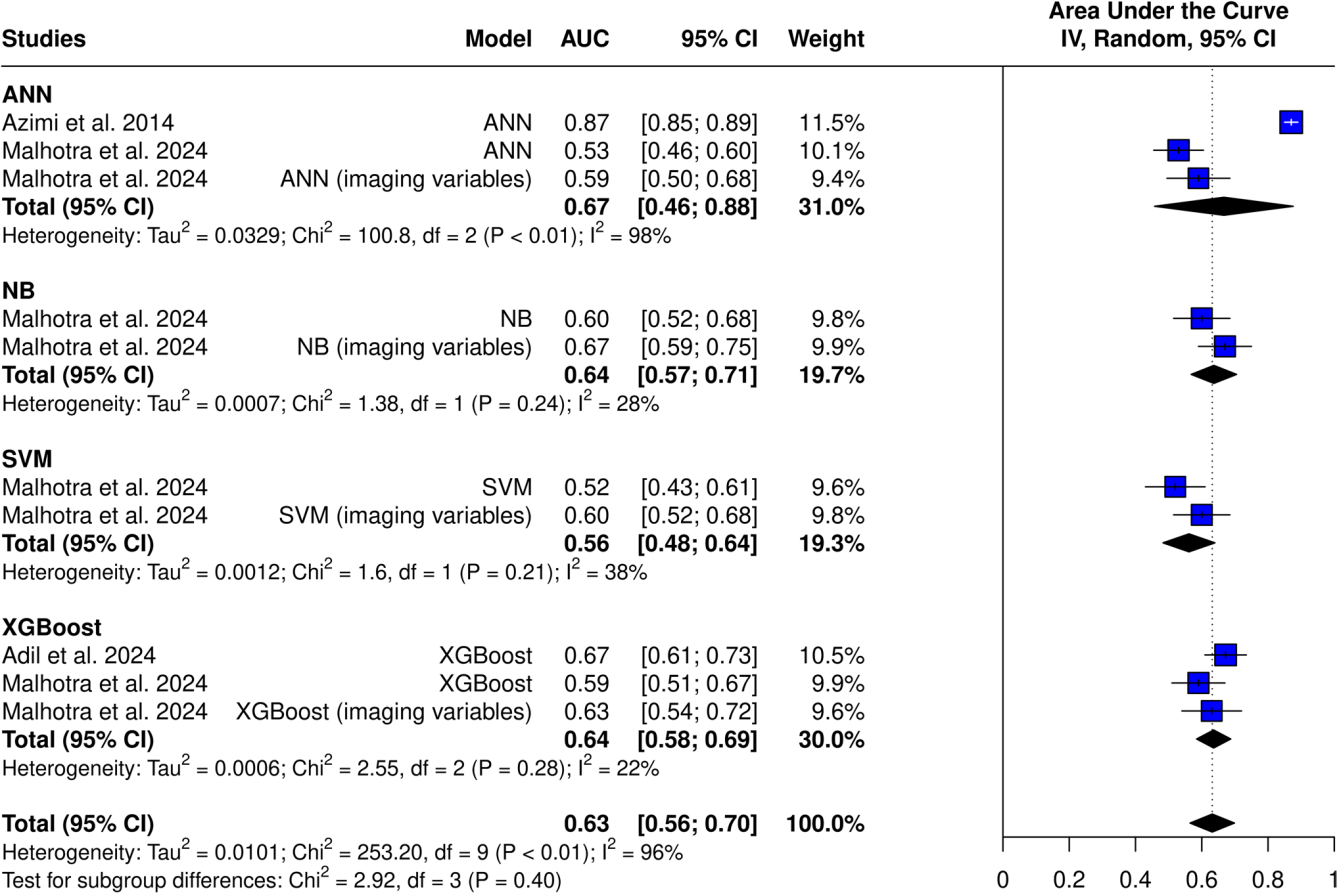
Overall pooled AUC from all ML models (ML model subgroups)

**Fig. 5:**
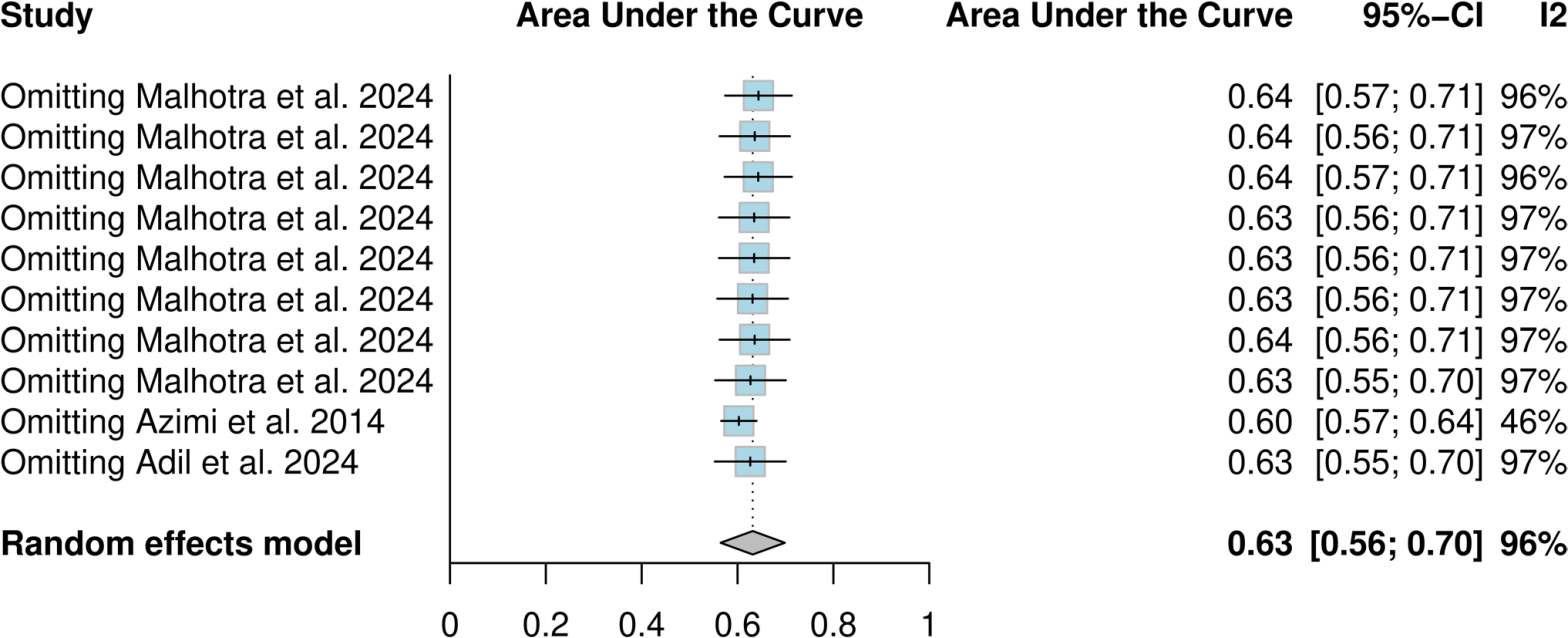
Leave-one-out analysis from all ML models

**Fig. 6:**
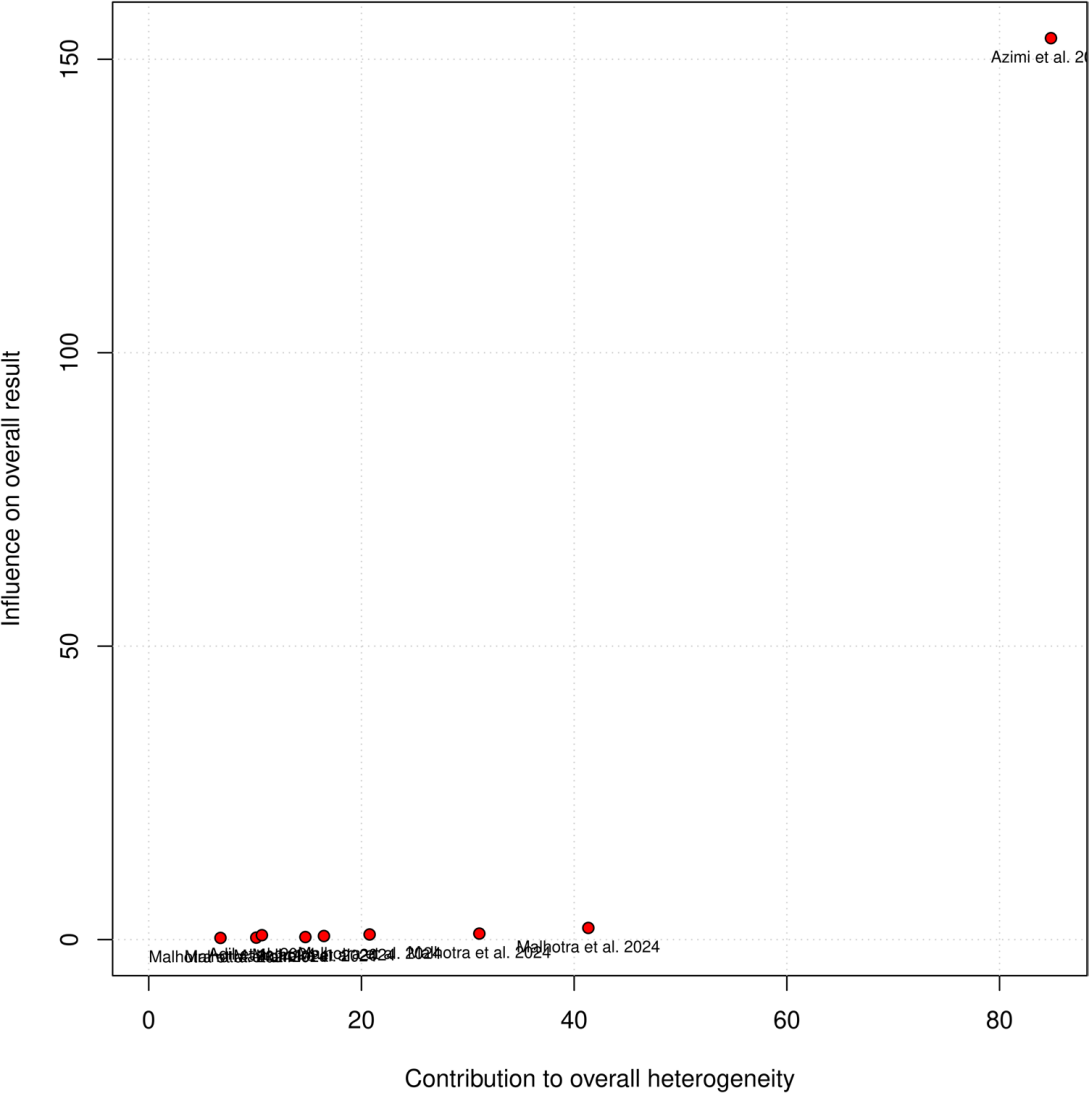
Baujat plot

**Fig. 7:**
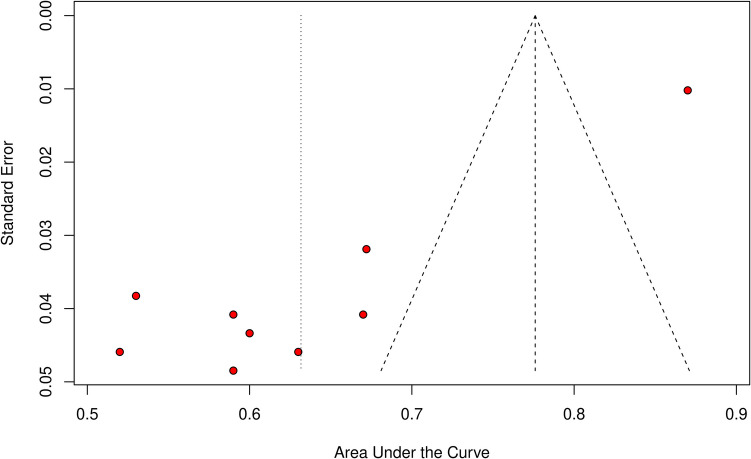
Funnel plot

Three studies and ten models from these studies were eligible for the AUC meta-analysis (Figs. 3 and 4). The overall AUC was 0.63 (95% CI 0.56–0.70). Heterogeneity was observed (I^2^ = 96%, *p* < 0.01). The subgroup with imaging variables achieved an AUC of 0.63 (95% CI 0.58–0.67), which was comparable to models without imaging variables—0.63 (95% CI 0.53–0.74). Azimi et al.’s ANN achieved the highest AUC of all models (0.87), while SVM from Malhotra et al. achieved the worst results (AUC = 0.52). Only after omitting Azimi et al., heterogeneity dropped to 46% (Fig. 5). This study was identified as an outlier in both the Baujat plot (Fig. 6) and influence diagnostics analysis. Publication bias was presented among the models; this was confirmed by funnel plot asymmetry (Fig. 7) and Egger’s test (*p* < 0.001).

**Fig. 8:**
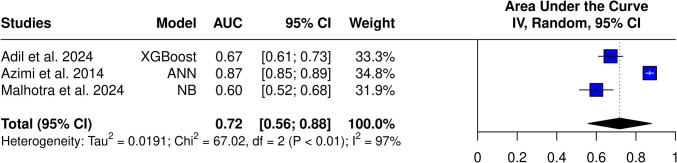
AUC of the best-performing models (without imaging variables)

Analysis of the best model from each study (without imaging features) yielded an AUC of 0.72 (95% CI 0.56–0.88) (Fig. 8). High heterogeneity was still present (I^2^ = 97%, *p* < 0.01). Analysis with models, which included imaging features, achieved an AUC of 0.74 (95% CI 0.61–0.88) without changing high heterogeneity (I^2^ = 96%, *p *< 0.01).

**Fig. 9:**
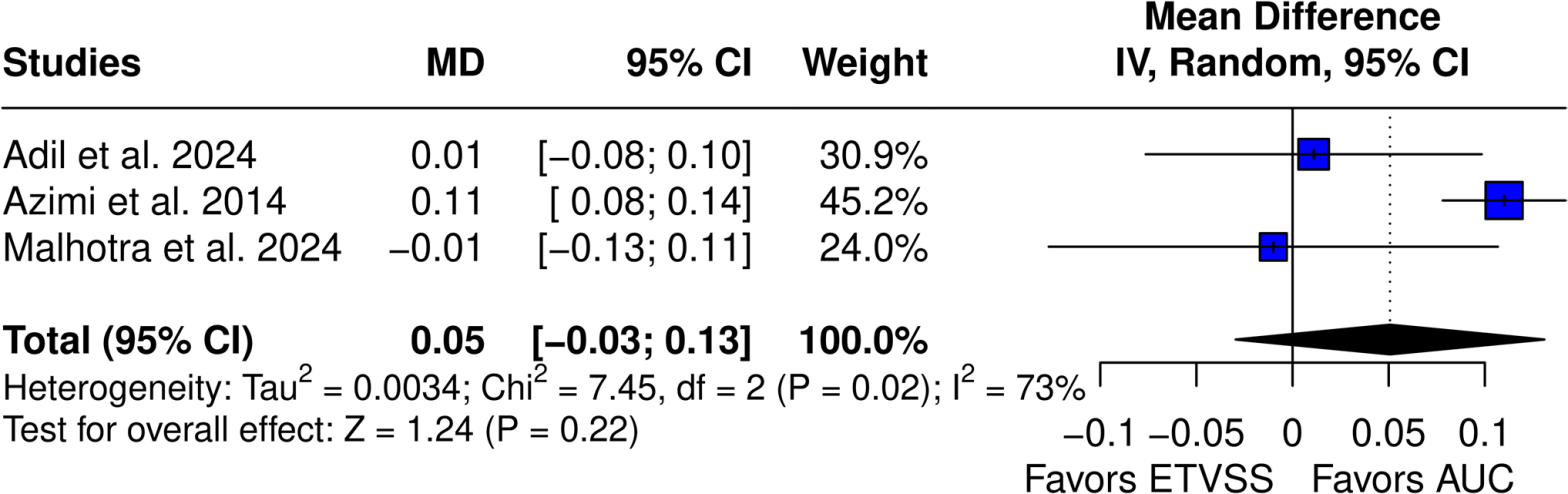
Mean difference of AUC between ML with imaging models vs. ETVSS

The mean difference of ML models compared to ETVSS was not significant (MD 0.03 (95% CI −0.08–0.13), *p* = 0.65), and high heterogeneity was observed (I^2^ = 84%, *p* < 0.01). With imaging variables, models did not achieve a significant difference (MD 0.05 (95% CI −0.03–0.13), *p* = 0.22) (Fig. 9).

**Fig. 10:**
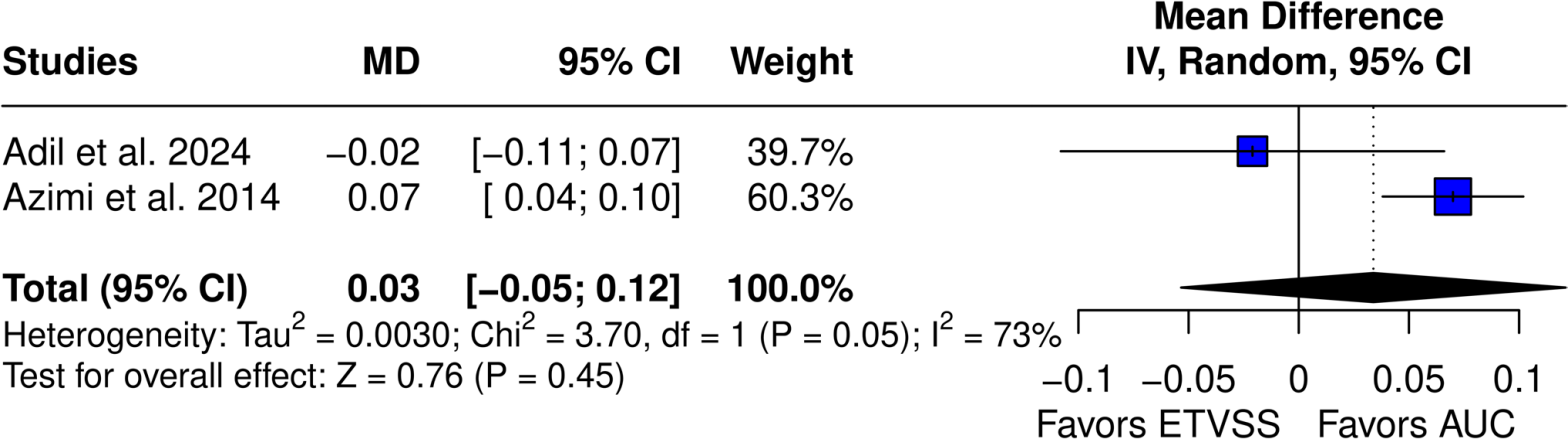
Comparison of ML vs. ETVSS models

When compared to LR models, ML algorithms did not achieve a statistically significant difference (MD 0.03 (95% CI −0.05–0.12), *p* = 0.45) (Fig. 10). High I^2^ was observed (73%).

### Narrative description of the included studies

Azimi and colleagues published the first study on the application of ML to the prediction of ETV success in 2014. The authors developed an ANN (multilayer perceptron), which was validated on a dataset with 168 patients in the study and compared to the LR model. Besides LR, the authors evaluated ANN to ETVSS and the CURE Children’s Hospital of Uganda (CCHU) ETV Success Score. ANN was developed with a total of seven variables: age of the patient, sex, body mass, hydrocephalus etiology and type, presence of choroid plexus cauterization intervention, and shunts in the patient’s history. The LR model was developed on the same prediction variables. The dataset was split in a 50%:25%:25% ratio for training, validation, and testing. ANN achieved 95.1% (95% CI 93.7–97.6%) accuracy, an AUC of 0.87 (95% CI 0.85–0.89), and 41.2 (95% CI 36.1–47.2) in the Hosmer–Lemeshow test. All three of these results were significantly higher when compared to LR, CCHU ETV, and ETVSS (*p *< 0.001). ANN also achieved 45% specificity, a positive predictive value of 69%, and a negative predictive value of 89%. Meanwhile, LR achieved an AUC of 0.80 (95% CI 0.75–0.80), ETVSS of 0.76 (95% CI 0.71–0.76), and CCHU ETV of 0.78 (95% CI 0.71–0.78).

The most important variables for prediction were etiology (AUC 0.68, *p* = 0.01), age (AUC 0.65, *p* = 0.02), choroid plexus cauterization procedure (AUC 0.64, *p* = 0.03), previous shunt history (AUC 0.63, *p* = 0.04), and type of hydrocephalus (AUC 0.60, *p* = 0.046). Patient sex (AUC 0.49, *p* = 0.84) and body weight (AUC 0.46, *p* = 0.79) were not statistically significant for prediction performance.

Adil and colleagues performed the biggest study, in terms of patient size, investigating the effectiveness of multiple models for ETV success prognosis. Their study included 2047 patients (of whom 1261 had successful ETV), and 15% of the dataset was used for testing. Notably, their study developed five different ML models for ETV prediction: SVM, RF, k-NN, XGBoost, and NB, which were compared to traditional LR and ETVSS prognosis. ML model variables included the patient’s age, etiology of the hydrocephalus, and presence of shunts in the patient’s history.

During ML model validation, models achieved AUC as follows: SVM (0.536), RF (0.658), k-NN (0.661), XGBoost (0.683), and NB (0.666). Both LR and ETVSS achieved a superior AUC of 0.693. For the final testing sets, XGBoost and LR were selected and compared to ETVSS. Again, LR achieved an AUC of 0.693 (95% CI 0.633–0.754), while XGBoost was 0.672 (95% CI 0.609–0.734). Both were insensibly higher when compared to the original ETVSS—0.661 (95% CI 0.600–0.722). Additional diagnostic results were as follows: sensitivity (0.332 vs. 0.316 vs. 0.284), specificity (0.847 vs. 0.831 vs. 0.881), PPV (0.778 vs. 0.750 vs. 0.794), and NPV (0.441 vs. 0.430 vs. 0.433) for LR, XGBoost, and ETVSS, respectively.

Malhotra et al. conducted a study on 752 patients (185 with ETV failure) to assess whether the ETVSS can outperform ML models. Additionally, they wanted to evaluate the effectiveness of the predictive models with additional imaging data for both ETVSS and ML algorithms. Their trial included 14 North American pediatric neurosurgery centers. Notably, authors excluded patients with previous unsuccessful ETV (surgery revision), ETV with choroid plexus cauterization, and previous shunts. Therefore, their prediction model was based only on demographic characteristics of corrected age and hydrocephalus etiology. For the models with image features, the authors extracted the frontal occipital horn ratio and maximal third ventricular width for model development. The authors used four different machine learning algorithms: NB, XGBoost, ANN, and SVM. Fivefold cross-validation was used for model development, and the dataset was split, 70% for training and 30% for model testing.

Without the imaging features, ETVSS showed the highest AUC of all models: 0.68 (95% CI 0.60–0.76). Similarly, ETVSS showed the highest AUC for prediction with two imaging features—0.68 (95% CI 0.59–0.76). NB was the best-performing model and achieved an AUC of 0.60 (95% CI 0.52–0.69) and 0.67 (95% CI 0.59–0.75) for classification without and with image-aided prediction, respectively. Other ML models' AUC results were as follows: XGBoost 0.59 (95% CI 0.51–0.67) and 0.63 (95% CI 0.54–0.72); ANN 0.53 (95% CI 0.46–0.61) and 0.59 (95% CI 0.49–0.68); and SVM 0.52 (95% CI 0.43–0.61) and 0.60 (95% CI 0.51–0.68), for classic prediction without, and with imaging features, respectively.

Finally, Masoudi et al. conducted a study testing a neural network model on 120 patients from Iran. Eight variables were used as predictors: patient’s age, bifrontal angle, callosal angle, subdural hygroma, bicaudate index, third ventricle width, temporal horn width, and frontal horn width. Again, the authors applied fivefold cross-validation for algorithm testing. The testing set was performed on 30% of patients. ANN achieved an accuracy of 0.859 and an AUC of 0.913. Next, they built a simplified model with just four features: patient’s age, bifrontal angle, temporal horn width, and frontal horn width. ANN achieved an accuracy of 0.84 and an AUC of 0.858, which were superior to the LR model with the same four variables (0.80 accuracy and AUC of 0.819).

## Discussion

This meta-analysis intended to analyze the effectiveness of ML models for the predictive modeling of ETV success. ETV became a commonly used procedure for hydrocephalus treatment, along with ventriculoperitoneal shunting. ETV’s success rate is around 80%; therefore, strong predictive models are needed [2]. However, developing a good model with a high discrimination rate is challenging due to the multifactorial nature of hydrocephalus, which includes patient-specific, surgical, and procedural variables. ML is becoming more and more present in the areas of neurological surgery [31]. Evaluation of ML effectiveness in traditional ETVSS and LR models becomes more prevalent in the literature. By conducting this paper, authors aimed to synthesize current knowledge on this subject and propose limitations, strengths, and future solutions.

Studies applied various machine learning techniques, including ANN, XGBoost, SVM, and RF. The performance between different models varied highly. The overall pooled AUC was found to be 0.63 (95% CI 0.56–0.70), which, according to the general interpretation of AUC values, reflects a moderate predictive ability for ETV success [20]. High heterogeneity was observed—the best model achieved an AUC of 0.87, while the weakest was only 0.52, which is comparable to random guessing.

The observed heterogeneity in the AUC values may be due to various factors, including differences in the baseline data among the included patients, types of algorithms used, validation techniques, model optimization and training, and inclusion of additional variables. ANN models achieved the highest AUC of 0.67, suggesting potential superiority over other ML techniques. On the other hand, SVM algorithms achieved an AUC of only 0.56, indicating that not all ML techniques may lead to highly accurate results.

High heterogeneity between the studies (I^2^ > 90%) could be explained by different predictive performance, which heavily relies on numerous factors, such as demographics of the patients, etiology of the hydrocephalus, inclusion of imaging variables for the prediction, testing set size, hyperparameter tuning, validation method, and ETVSS.

Malhotra and colleagues indicated that the inclusion of imaging variables slightly improves the ML predictive performance; however, this improvement did not significantly change prediction performance, which remained under an AUC of < 0.70 [27]. The improvement was not substantial enough to make imaging features a consistent predictor across all studies, reflecting the need for the inclusion of more variables for ETV success prediction.

Compared to traditional prognosis methods, including ETVSS and LR, their results remained statistically insignificant, and there were no impactful mean difference in AUC between ML and conventional prognostic methods. Pairwise meta-analysis did not show a significant difference in the AUC between ML and ETVSS (mean difference 0.03, 95% CI −0.08 to 0.13, *p *= 0.65) or between ML and LR models (mean difference 0.03, 95% CI −0.05 to 0.12, *p* = 0.45). Therefore, it is safe to assume that the current development of ML techniques has not brought sophisticated models for accurate ETV success prediction, and future developments are needed in this area. ML at its current state does not outperform traditional techniques, and all currently available methods show at most moderate clinical application.

Regarding potential clinical application, the currently available evidence in the literature indeed shows future potential for ML in this area; however, there are several important limitations that need to be resolved before ML will be integrated for prognosis in neurosurgical operations. Firstly, there was heterogeneity in the patient populations (hydrocephalus etiology, age, sex), and some studies excluded patients with previous shunts. Therefore, future models should be adjusted and adapted for highly heterogeneous clinical applications. AUC with confidence intervals was not available in the Masoudi et al. study, which authors had to exclude from the analysis [30]. Overfitting of the machine learning models was not adjusted in some studies [32]. Missing data were also present in some studies, limiting the inclusion of more predictive variables for the analysis [33]. In some studies, the sample size was relatively small; therefore, ML could reach overoptimistic results in the small dataset [28, 30].

Furthermore, several quality assessment concerns were found among the included studies. None of the studies was graded as low risk of bias; all of them had either moderate or serious concerns. There was a high risk of bias in some studies, mainly due to confounding factors, participant selection, and reporting of results. Future studies must carefully consider these areas of bias to provide reliable results of the ML prediction models for ETV success. This aspect is crucial for potential clinical application.

Additionally, limited external validation was performed on the studies. Ideally, models should be validated on datasets from unseen data [34]. Economic cost-effectiveness was not performed due to insufficient data from the studies. This systematic review is also limited by a relatively small number of papers included in the synthesis and a few different ML models. The number of CNN, DL, and RF models was scarce—these techniques should be explored for model performance in future trials.

To minimize current limitations, several strategies are proposed. Firstly, more multicenter trials are needed in the future; single-center analyses may lead to overoptimistic results and potential bias in the analysis. Validation techniques mostly included fivefold cross and split techniques; therefore, other approaches (i.e., tenfold cross) could be tested or even compared to currently applied methods to assess whether they impact the model performance [35].

Furthermore, the integration of more variables (i.e., prepontine adhesions, cerebral aqueduct morphology, presence of residual membranes, arachnoid adhesions, or optic nerve sheath diameter) might potentially influence the impact of ML effectiveness [27–30, 36–38]. The inclusion of imaging variables showed an increase in predictive model performance with just two variables [27]. Future trials could integrate multiple imaging parameters to enhance the prediction of unsuccessful operations.

Finally, standardization of outcome reporting is needed, as studies reported a high variety in methodology reporting. This would reduce heterogeneity and provide the possibility to more accurately compare the effectiveness of different ML techniques. Examples of such protocols include APPRAISE-AI and TRIPOD [39, 40].

## Conclusions

 ML models show moderate potential for predicting ETV success and do not outperform traditional tools like ETVSS and LR models in clinical application. High heterogeneity and methodological limitations suggest that further research is needed to optimize and validate ML algorithms. Larger, multicenter studies with improved reporting and external validation are essential for enhancing the generalizability and clinical utility of these models.

## Supplementary information

Below is the link to the electronic supplementary material.

Supplementary file (PDF 50.2 KB)

## Data Availability

No datasets were generated or analysed during the current study.
