# Supplementary material for: Machine learning for endoscopic third ventriculostomy success prediction—a systematic review and meta-analysis
Source: Childs Nerv Syst. 2025 Sep 29;41(1):297. doi: 10.1007/s00381-025-06962-7 (PMC12479695; doi:10.1007/s00381-025-06962-7)
Supplement: Supplementary file 1 — Supplementary file (PDF 50.2 KB) [file 381_2025_6962_MOESM1_ESM.pdf]

Scopus
TITLE-ABS-KEY (((endoscopic AND third AND ventriculostomy AND success AND score) OR etv OR etvss OR (endoscopic AND third AND ventriculostomy) OR ventriculostomy OR (third AND ventricle)) AND ((artificial AND intelligence) OR (convolutional AND neural AND network) OR cnn OR (deep AND learning) OR (machine AND learning) OR (decision AND tree) OR (neural AND network) OR (support AND vector AND machine) OR (k-means) OR (artificial AND neural) OR (reinforcement AND learning)))

WOS
((Endoscopic Third Ventriculostomy Success Score) OR ETV OR ETVSS OR (Endoscopic Third Ventriculostomy) OR Ventriculostomy OR (Third Ventricle)) AND ((artificial AND intelligence) OR (convolutional AND neural AND network) OR cnn OR (deep AND learning) OR (machine AND learning) OR (decision AND tree) OR (neural AND network) OR (support AND vector AND machine) OR (k-means) OR (artificial AND neural) OR (reinforcement AND learning))

Cochrane

((Endoscopic Third Ventriculostomy Success Score) OR ETV OR ETVSS OR (Endoscopic Third Ventriculostomy) OR Ventriculostomy OR (Third Ventricle)) AND ((artificial AND intelligence) OR (convolutional AND neural AND network) OR cnn OR (deep AND learning) OR (machine AND learning) OR (decision AND tree) OR (neural AND network) OR (support AND vector AND machine) OR (k-means) OR (artificial AND neural) OR (reinforcement AND learning)) in Title Abstract Keyword

Embase
('endoscopic third ventriculostomy success score'/exp OR 'endoscopic third ventriculostomy success score' OR (endoscopic AND third AND ('ventriculostomy'/exp OR ventriculostomy) AND ('success'/exp OR success) AND ('score'/exp OR score)) OR etv OR etvss OR 'endoscopic third ventriculostomy'/exp OR 'endoscopic third ventriculostomy' OR (endoscopic AND third AND ('ventriculostomy'/exp OR ventriculostomy)) OR 'ventriculostomy'/exp OR ventriculostomy OR 'third ventricle'/exp OR 'third ventricle' OR (third AND ventricle)) AND (artificial AND ('intelligence'/exp OR intelligence) OR (convolutional AND neural AND ('network'/exp OR network)) OR cnn OR (deep AND ('learning'/exp OR learning)) OR (('machine'/exp OR machine) AND ('learning'/exp OR learning)) OR (('decision'/exp OR decision) AND ('tree'/exp OR tree)) OR (neural AND ('network'/exp OR network)) OR (('support'/exp OR support) AND ('vector'/exp OR vector) AND ('machine'/exp OR machine)) OR 'k means'/exp OR 'k means' OR (artificial AND neural) OR (('reinforcement'/exp OR reinforcement) AND ('learning'/exp OR learning)))

PubMED

((("ventriculostomy"[MeSH Terms] OR "ventriculostomy"[All Fields] OR ("endoscopic"[All Fields] AND "third"[All Fields] AND "ventriculostomy"[All Fields]) OR "endoscopic third ventriculostomy"[All Fields]) AND ("success"[All Fields] OR "successes"[All Fields] OR "successful"[All Fields]) AND ("score"[All Fields] OR "score s"[All Fields] OR "scored"[All Fields] OR "scores"[All Fields] OR "scoring"[All Fields] OR "scorings"[All Fields])) OR "ETV"[All Fields] OR "ETVSS"[All Fields] OR ("ventriculostomy"[MeSH Terms] OR "ventriculostomy"[All Fields] OR ("endoscopic"[All Fields] AND "third"[All Fields] AND "ventriculostomy"[All Fields]) OR "endoscopic third ventriculostomy"[All Fields]) OR ("ventriculostomy"[MeSH Terms] OR "ventriculostomy"[All Fields] OR "ventriculostomies"[All Fields]) OR ("third ventricle"[MeSH Terms] OR ("third"[All Fields] AND "ventricle"[All Fields]) OR "third ventricle"[All Fields])) AND ((("artificial"[All Fields] OR "artificially"[All Fields]) AND ("intelligence"[MeSH Terms] OR "intelligence"[All Fields] OR "intelligences"[All Fields] OR "intelligent"[All Fields] OR "intelligently"[All Fields] OR "intelligibilities"[All Fields] OR "intelligibility"[All Fields] OR "intelligible"[All Fields])) OR (("convolute"[All Fields] OR "convoluted"[All Fields] OR "convolutes"[All Fields] OR "convoluting"[All Fields] OR "convolution"[All Fields] OR "convolutional"[All Fields] OR "convolutions"[All Fields] OR "convolutive"[All Fields]) AND ("neural"[All Fields] OR "neuralization"[All Fields] OR "neuralize"[All Fields] OR "neuralized"[All Fields] OR "neuralizes"[All Fields] OR "neuralizing"[All Fields] OR "neurally"[All Fields]) AND ("network"[All Fields] OR "network s"[All Fields] OR "networked"[All Fields] OR "networker"[All Fields] OR "networkers"[All Fields] OR "networking"[All Fields] OR "networks"[All Fields])) OR "cnn"[All Fields] OR ("deep"[All Fields] AND ("learning"[MeSH Terms] OR "learning"[All Fields] OR "learn"[All Fields] OR "learned"[All Fields] OR "learning s"[All Fields] OR "learnings"[All Fields] OR "learns"[All Fields])) OR (("machine"[All Fields] OR "machine s"[All Fields] OR "machines"[All Fields]) AND ("learning"[MeSH Terms] OR "learning"[All Fields] OR "learn"[All Fields] OR "learned"[All Fields] OR "learning s"[All Fields] OR "learnings"[All Fields] OR "learns"[All Fields])) OR (("decision"[All Fields] OR "decision s"[All Fields] OR "decisions"[All Fields] OR "decisive"[All Fields] OR "decisively"[All Fields]) AND ("trees"[MeSH Terms] OR "trees"[All Fields] OR "tree"[All Fields])) OR (("neural"[All Fields] OR "neuralization"[All Fields] OR "neuralize"[All Fields] OR "neuralized"[All Fields] OR "neuralizes"[All Fields] OR "neuralizing"[All Fields] OR "neurally"[All Fields]) AND ("network"[All Fields] OR "network s"[All Fields] OR "networked"[All Fields] OR "networker"[All Fields] OR "networkers"[All Fields] OR "networking"[All Fields] OR "networks"[All Fields])) OR (("support"[All Fields] OR "support s"[All Fields] OR "supported"[All Fields] OR "supporter"[All Fields] OR "supporter s"[All Fields] OR "supporters"[All Fields] OR "supporting"[All Fields] OR "supportive"[All Fields] OR "supportiveness"[All Fields] OR "supports"[All Fields]) AND ("genetic vectors"[MeSH Terms] OR ("genetic"[All Fields] AND "vectors"[All Fields]) OR "genetic vectors"[All Fields] OR "vector"[All Fields] OR "vectors"[All Fields] OR "vector s"[All Fields] OR "vectored"[All Fields] OR "vectoring"[All Fields] OR "vectorization"[All Fields] OR "vectorize"[All Fields] OR "vectorized"[All Fields] OR "vectorizing"[All Fields]) AND ("machine"[All Fields] OR "machine s"[All Fields] OR "machines"[All Fields])) OR "k-means"[All Fields] OR (("artificial"[All Fields] OR "artificially"[All Fields]) AND ("neural"[All Fields] OR "neuralization"[All Fields] OR "neuralize"[All Fields] OR "neuralized"[All Fields] OR "neuralizes"[All Fields] OR "neuralizing"[All Fields] OR "neurally"[All Fields])) OR (("reinforce"[All Fields] OR "reinforced"[All Fields] OR "reinforcement, psychology"[MeSH

Terms] OR ("reinforcement"[All Fields] AND "psychology"[All Fields]) OR "psychology reinforcement"[All Fields] OR "reinforcement"[All Fields] OR "reinforcements"[All Fields] OR "reinforcer"[All Fields] OR "reinforcer s"[All Fields] OR "reinforcers"[All Fields] OR "reinforces"[All Fields] OR "reinforcing"[All Fields]) AND ("learning"[MeSH Terms] OR "learning"[All Fields] OR "learn"[All Fields] OR "learned"[All Fields] OR "learning s"[All Fields] OR "learnings"[All Fields] OR "learns"[All Fields])))
